# Green Synthesis of Silver Nanoparticles with Antibacterial Activity Using Various Medicinal Plant Extracts: Morphology and Antibacterial Efficacy

**DOI:** 10.3390/nano11041005

**Published:** 2021-04-14

**Authors:** Aneta Salayová, Zdenka Bedlovičová, Nina Daneu, Matej Baláž, Zdenka Lukáčová Bujňáková, Ľudmila Balážová, Ľudmila Tkáčiková

**Affiliations:** 1 Department of Chemistry, Biochemistry and Biophysics, University of Veterinary Medicine and Pharmacy, Komenského 73, 041 81 Košice, Slovakia; zdenka.bedlovicova@uvlf.sk; 2 Advanced Materials Department, Jožef Stefan Institute, Jamova cesta 39, 1000 Ljubljana, Slovenia; nina.daneu@ijs.si; 3 Department of Mechanochemistry, Institute of Geotechnics, Slovak Academy of Sciences, Watsonova 45, 040 01 Košice, Slovakia; balazm@saske.sk (M.B.); bujnakova@saske.sk (Z.L.B.); 4 Department of Pharmaceutical Technology, Pharmacognosy and Botany, University of Veterinary Medicine and Pharmacy, Komenského 73, 041 81 Košice, Slovakia; ludmila.balazova@uvlf.sk; 5 Department of Microbiology and Immunology, University of Veterinary Medicine and Pharmacy, Komenského 73, 041 81 Košice, Slovakia; ludmila.tkacikova@uvlf.sk

**Keywords:** AgNP biosynthesis, antibacterial activity, green synthesis, antioxidant activity, plant extracts, characterisation

## Abstract

A green synthetic route for the production of silver nanoparticles (AgNPs) using five different aqueous plant extracts, namely, *Berberis vulgaris*, *Brassica nigra*, *Capsella bursa-pastoris*, *Lavandula angustifolia* and *Origanum vulgare*, was investigated in this study. The present work demonstrates the influence of plant extract composition (antioxidant and total phenolic content) on the size and morphology of the produced AgNPs. The biosynthetic procedure was rapid and simple and was easily monitored via colour changes and ultraviolet and visible (UV-Vis) spectroscopy. Subsequently, measurement of zeta potential (ZP), photon cross-correlation spectroscopy (PCCS), X-ray diffraction (XRD), Fourier-transform infrared spectroscopy (FTIR), transmission electron microscopy (TEM) and selected area electron diffraction (SAED) analysis were employed to characterise the as-synthesised nanoparticles. The XRD investigation confirmed the presence of Ag^0^ in the nanoparticles, and interactions between the bioactive compounds of the plants and the produced AgNPs were evident in the FTIR spectra. TEM indicated that the nanoparticles exhibited a bimodal size distribution, with the smaller particles being spherical and the larger having a truncated octahedron shape. In addition, the antimicrobial activity of the AgNPs was tested against five bacterial strains. All synthesised nanoparticles exhibited enhanced antimicrobial activity at a precursor concentration of 5 mM compared to the control substance, gentamicin sulphate, with the best results observed for AgNPs prepared with *B. nigra* and *L. angustifolia* extracts.

## 1. Introduction

Scientists around the world are focusing on nano-bioscience due to its potential in several applications, one of them being their potential to combat bacterial resistance. As a result of the incorrect and overuse of antibiotics, bacterial resistance has become a world-wide problem in the treatment of infectious diseases. Due to the need for new antimicrobial agents that are able to kill or inhibit the growth of a wide range of microbes, the use of nanotechnology in the development of effective antimicrobials is a new promising alternative. The antimicrobial activity of nanoparticles (NPs) results from a very different mechanism compared to antibiotics. It is hypothesised that the surface area of the NPs is a key component in their activity, but it varies from one type of NP to another [1]. The antimicrobial activity of silver is already well established, but research into nano-size silver (<100 nm) has increased due to its multidisciplinary application and extraordinary defence against a broad spectrum of bacteria, including both Gram-negative and Gram-positive strains [2]. The most important physicochemical parameters affecting the antimicrobial potential of AgNPs include size, shape, surface charge, concentration and colloidal state. Mode of action may be associated with various mechanisms: in terms of adhesion to microbial cells, penetration inside the cells, ROS (reactive oxygen species) and free radical generation, or modulation of microbial signal transduction pathways [3,4]. AgNPs are currently used in the textile industry and in wastewater treatment, but they are used most commonly in biomedical applications [5,6], such as bio-sensing, imaging and drug delivery, and as antimicrobial [7], anticancer and larvicidal agents [8]. At present, interest is focused on the antiviral activity of AgNPs in view of the COVID-19 pandemic [9,10].

There are various methods for the synthesis of AgNPs, belonging to chemical [11], physical [12,13] and biological ones [14,15,16]. Furthermore, AgNPs can be prepared in a sustainable manner, which is beneficial due to its simplicity, low cost and eco-friendly manner, making it harmless for both human and animal health [17,18,19,20]. Generally, “green” bio-synthesis of AgNPs can be aided using bacteria [8,21,22], fungi [23], algae [24] or plants (currently being used extensively) [25] as intracellular or extracellular sources for the reduction of Ag^+^ to Ag^0^. Generally, the mechanism of bio-reduction can be divided into the three main steps: (1) silver ion reduction and nucleation, (2) the growing step and aggregation, and (3) capping and stabilisation in the terminal step. The crucial role is always played by the phytochemicals of the plant, namely, secondary metabolites such as sugars, polyphenols, proteins, phenolic acids, ketones, terpenoids, amides, etc. Moreover, in the majority of cases, a reducing agent from the plant extract plays a role as both the capping and stabilising agents [26,27,28].

In this study, we employed medicinal plants used widely in folk medicine from a diverse range of families, including Lamiaceae (*Lavandula angustifolia* and *Origanum vulgare*), Brassicaceae (*Brassica nigra* and *Capsella bursa-pastoris*) and Berberidaceae (*Berberis vulgaris*). Regarding the plants used in our study, the successful synthesis of AgNPs using extracts of *B. nigra* [29], *O. vulgare* [30,31,32,33], *B. vulgaris* leaf and root aqueous extract [34], or *L. angustifolia* flowers extract [35,36,37] has been reported. To the best of our knowledge, the extracts from *B. vulgaris* roots, *L. angustifolia* leaves and *C. bursa-pastoris* leaves for the preparation of AgNPs have not been reported. Table 1 shows a literature review of the main findings of green AgNP synthesis using the selected plants and a comparison with the main results obtained in our study. These results are also discussed later in corresponding subsections.

*Lavandula* (commonly lavender) has been used for centuries in cosmetics but is also interesting for its antifungal [38,39] and antibacterial properties [40,41]. *O. vulgare* is beneficial for human health and is quite common in nature, containing substances with antibacterial and antioxidant activity [42,43]. *C. bursa-pastoris* is one of the plants commonly used in traditional medicine and exhibits antimicrobial, anti-inflammatory, antioxidant, cardioprotective, increasing reproductive efficiency, anticancer, hepatoprotective, sedative and other pharmacological effects [44]. *B. nigra* also has important medicinal uses, such as in the treatment of rheumatism and joint pains, neuralgia and spasms, alopecia, epilepsy and throat tumours and as a laxative [45]. *B. vulgaris* has been used in Chinese medicine for thousands of years and has been studied for its antihistaminic and, nowadays, its anticholinergic activity [46] and activity against cardiovascular disorders [47]. *B. vulgaris* also exhibits a glucose-lowering effect and has been used in the treatment of diabetes [48,49], as are *O. vulgare* [50] and *B. nigra* [51]. These medicinal plants also result in the well-reported antioxidant behaviour, etc. The aims of this study are to compare the antioxidant activity of each plant extract and to predict if it is able to reduce silver ions during the AgNP biosynthesis.

In addition, as different parts of the plants (leaves, seeds and roots) were used, this work offers a comparison of the produced NPs in terms of their size, optical properties and antimicrobial activity in one summarising study. The aim of this study is to synthesise AgNPs in an eco-friendly manner using different plant extracts prepared under mild conditions to avoid the loss of thermally unstable compounds. Successfully prepared AgNPs were characterised, and their antibacterial activity in vitro was evaluated. The novelty of this study lies in the utilisation of a rich plethora of plant extracts and a simplified synthetic approach for the preparation of AgNPs directly in the UV-Vis cuvette, which enables immediate monitoring of the reaction progress.

## 2. Materials and Methods

### 2.1. Materials and Chemicals

*Brassica nigra* seeds (Benkor, Velký Borek, Czech Republic), *Capsella bursa-pastoris* leaves (Hanus, Nitra, Slovakia) and *Lavandula angustifolia* leaves (Juvamed, Rimavská Sobota, Slovakia) were purchased as commercial products. *Origanum vulgare* leaves were collected in summer from the meadow at the University of Veterinary Medicine and Pharmacy campus in Košice, Slovakia. *Berberis vulgaris* root was obtained in spring from a private garden in Byster, Slovakia. Collected leaves and roots were dried up to constant weight in the dark at room temperature. Silver nitrate (AgNO_3_, 99.9%, Mikrochem, Pezinok, Slovakia), 2,2-diphenyl-1-picrylhydrazyl (DPPH) (Sigma Aldrich, St. Louis, MO, USA), Folin–Ciocalteau reagent (Sigma Aldrich, St. Louis, MO, USA), methanol (p.a. 99.5%, Mikrochem, Slovakia), sodium carbonate (>99%, Mikrochem, Pezinok, Slovakia), gallic acid (97.5–102.5%, Sigma Aldrich, St. Louis, MO, USA) and gentamicin sulphate (Sigma-Aldrich, St. Louis, MO, USA) were used without further purification.

### 2.2. Antioxidant Activity—DPPH Method

The antioxidant activities were determined by DPPH (2,2-diphenyl-1-picrylhydrazyl) assay according to the methodology described by Brand-Williams [52]. Briefly, 100 μL of plant extract was added to 0.9 mL methanol and 2 mL of DPPH solution (0.1 mM) and the mixtures were incubated in the dark for 30 min at room temperature. The blank was prepared in parallel from 1 mL of methanol and 2 mL of DPPH solution. The absorbance was measured at 517 nm using UV-Vis spectrophotometer Cary 60 (Agilent Technologies, Santa Clara, CA, USA), and the scavenging activity percentage (AA%) was determined according to Equation (1):

(1) $$\mathrm{AA}\%=100\times \left[\frac{A_{\mathrm{blank}}\;-A_{\mathrm{sample}}}{A_{\mathrm{blank}}}\right]$$

where A_sample_ is the absorbance of the plant extract with DPPH solution and A_blank_ is the negative control (methanol replacing the plant extract). The data were expressed as an average of triplicates ± standard deviation (SD).

### 2.3. Total Phenolic Content (TPC)

Detection of the TPC in plant extracts was estimated by the Folin–Ciocalteau colorimetric method [53]. Briefly, the reaction mixture was prepared with 20 μL of each extract or standard solution of gallic acid (50–1500 mg/L), 1.6 mL of freshly distilled water and 0.1 mL of Folin–Ciocalteau’s reagent. In the blank sample, 20 μL of water was used instead of plant extracts. After mixing, the samples were left for 5 min at room temperature and 0.3 mL of a 10% solution of sodium carbonate (Na_2_CO_3_) was added. The mixture was left in the dark at room temperature for 2 h, and the absorbance at 765 nm was measured spectrophotometrically using UV-Vis spectrophotometer Cary 60 (Agilent Technologies, Santa Clara, CA, USA). The equation obtained from the gallic acid calibration curve was y = 0.8789x + 0.0145 (R² = 0.9988). Total phenolic content is expressed in terms of mg gallic acid equivalents (GAE/mL). All results are expressed as mean ± SD (*n* = 3).

### 2.4. Preparation of Plant Extracts

To obtain the plant extracts, the seeds, leaves and root barks were thinly chopped into small pieces and powdered well with the grinder. Following that, one gram of dried powdered parts of plants was suspended in 20 mL of freshly distilled water. The suspensions were mixed at room temperature for 2 h, the solid residues were removed by filtration using filter paper (Whatman No. 1) and filtrates were used for silver nanoparticles synthesis.

### 2.5. Biosynthesis of AgNPs

For the synthesis of silver nanoparticles, the plant extracts of *Berberis vulgaris* (for BV-AgNPs), *B. nigra* (for BN-AgNPs), *C. bursa-pastoris* (for CBP-AgNPs), *L. angustifolia* (for LA-AgNPs) and *O. vulgare* (for OV-AgNPs) were added to freshly prepared and heated aqueous silver nitrate solution to 90 °C (2 mM) or 80 °C (5 mM) at a ratio of 1:9.

Briefly, 2.7 mL of freshly prepared aqueous silver nitrate solution was heated up using 1 cm optical path length quartz cuvettes with Peltier heating equipment. A freshly prepared plant extract (0.3 mL) was added into the warm solution. The formation of AgNPs was observed visually by detecting colour changes and by monitoring UV-Vis spectra every minute. The heating and monitoring of reaction were stopped when the absorbance in the monitored region did not increase anymore.

For XRD and FTIR characterisation of nanoparticles, the reaction was scaled up and performed in a round bottom flask from 250 mL of AgNO_3_ solution (5 mM) and 27.7 mL plant extract with heating to 80 °C. The heating was removed after the corresponding time and the mixture was left to stand for 48 h. The resulting colloidal suspensions were centrifuged in a Eppendorf Centrifuge 5430 (Eppendorf, Hamburg, Germany) at 7830 rpm for 15 min, and the precipitate was washed two times with sterile distilled water. The purified pellets were dried at 45 °C.

### 2.6. Characterisation Techniques

The silver nanoparticles formation was monitored by UV-Vis spectrophotometer Cary 60 (Agilent Technologies, Santa Clara, CA, USA) with Peltier heating and with thermostatted cell holders. For grain size distribution of the nanosuspensions, the PCCS method utilising a Nanofox particle size analyser (Sympatec, Clausthal-Zellerfeld, Germany) was used. The analyses were performed using a dispersant with the refractive index of 1.33. The measurements were repeated three times for each sample. The ZP values were measured using a Zetasizer Nano ZS (Malvern, Malvern, Worcestershire, UK). The ZP values were obtained by applying the Helmholtz–Smoluchowski equation built into Malvern Zetasizer software. Measurements were also repeated three times for all samples. The size, shape and chemical composition of the nanoparticles and the capping agents were analysed with TEM. The suspensions were ultrasonically homogenised for 3 min in ultrasonic bath Emmi-30HC (EMAG, Mörfelden-Walldorf, Germany) operating at 40 kHz at room temperature. The suspensions were applied onto a lacey carbon 200 mesh nickel grids (SPI Supplies, West Chester, PA, USA) and dried. Prior to TEM analyses, the grids were coated with 3 nm layer of amorphous carbon (Gatan PECS 68s, Pleasanton, CA, USA) to improve surface electron conductivity and to prevent charging under the high-energy electron beam. TEM analyses were performed using a 200 kV microscope JEM 2100 (JEOL, Akishima, Tokyo, Japan) with LaB_6_ electron source and equipped with an energy-dispersive X-ray spectrometer (EDS) for chemical analyses. The average diameter of AgNPs was determined by encircling the particles and transforming their areas with irregular shape into circles with an equivalent surface. In each sample, more than 100 NPs were measured. The particle size analysis was performed using a dedicated microstructure analysis program Image Tool^TM^ (University of Texas Health Science Center, San Antonio, TX, USA). The XRD patterns of the solids obtained after centrifugation were recorded using a D8 Advance diffractometer (Bruker, Berlin, Germany) with CuKα (40 kV, 40 mA) radiation. All samples were scanned from 15° to 70° with steps at 0.06° and 10 s of counting time. FTIR spectra of the dried extracts and of the solids obtained after centrifugation were recorded with an infrared spectrometer Tensor 29 (Bruker, Berlin, Germany) using the attenuated total reflectance (ATR) method and in the range 4000–650 cm^−1^.

### 2.7. Antibacterial Activity Using Agar Well-Diffusion Assay

The tested microorganisms (*Staphylococcus aureus* CCM 4223, *Listeria monocytogenes* CCM 4699, *Escherichia coli* CCM 3988, *Salmonella enterica* ser. Typhimurium CCM 7205 and *Pseudomonas aeruginosa* CCM 3989) were obtained from the Czech collection of microorganisms (CCM, Brno, Czech Republic). Microbes were selected based on their pharmacological and clinical significance. Frozen glycerol stock cultures were maintained at −20 °C. In brief, bacteria were cultured aerobically at 37 °C in nutrient broth (Oxoid, Basingstoke, UK) with agitation or on standard plate count agar (Oxoid, Basingstoke, UK). Before the experimental use, the cultures were transferred to the liquid media and incubated for 24 h. The cultures were then subcultured in liquid media, incubated for 24 h and used as the source of inoculum for each experiment. The agar media was cooled to 42 °C after autoclaving, inoculated with liquid overnight bacterial culture to a cell density of 5 × 10^5^ CFU mL^−1^ and 20 mL of this inoculated agar uniformly spread on the solid media plates (90 mm diameter Petri dishes). Once the agar was solidified, the wells (5.5 mm diameter) were punched in the agar and filled with 50 μL of samples. The antibiotic gentamicin sulphate (10 mM) was used as a standard antibacterial agent against all microbes. After the incubation for 24 h at 37 °C, the plates were photographed and the inhibition zone diameter (IZD) was measured by the ImageJ software 1.53e (U. S. National Institutes of Health, Bethesda, MD, USA). The diameter of the clear zones were the mean values of 3 replicate tests. The antibacterial activity was calculated by applying Equation (2) [54]:

(2) $$\mathrm{RIZD}\;=\;\frac{{\mathrm{IZD}}_{\mathrm{sample}}–{\;\mathrm{IZD}}_{\mathrm{negative}\;\mathrm{control}}\;}{{\mathrm{IZD}}_{\mathrm{gentamicin}\;\mathrm{sulfate}\;}–{\;\mathrm{IZD}}_{\mathrm{negative}\;\mathrm{control}}}\times 100\;\;$$

where RIZD is the relative inhibition zone diameter (%) and IZD is the inhibition zone diameter (mm). Corresponding plant extracts were used as the negative control, and gentamicin sulphate (10 mM) was used as a positive control. All values are expressed as mean ± standard deviation.

### 2.8. Statistical Analysis

GraphPad Prism 5 (GraphPad Software, San Diego, CA, USA) was used for statistical analyses. One-way ANOVA test with Dunnett post hoc test was used to evaluate the data. The results are expressed as the mean ± standard deviation. Values of *p* < 0.05 (*), *p* < 0.01 (**) and *p* < 0.001 (***) were considered significant.

## 3. Results and Discussion

### 3.1. Characterisation of Plant Extracts

For a better comparison of individual aqueous plant extracts, TPC and antioxidant activity were determined using a DPPH assay. The results are summarised in Table 2. It was observed that the TPC in the aqueous plant extracts correlates with the antioxidant activity, determined using the DPPH method. The highest antioxidant and TPC value were found in the case of an extract of *O. vulgare*, and the lowest values were determined for *B. vulgaris* and *B. nigra*. The phenolic content is mostly responsible for antioxidant activity, and it was also found to be important for AgNP biosynthesis [55,56].

The antioxidant capacity of the studied plant extracts is in the range of 61.78% (*B. vulgaris*) and 94.98% (*O. vulgare*). Olteanu et al. [57] prepared AgNPs from lavender and oregano extracts and reported antioxidant activities of 54.12 ± 1.18% and 33.53 ± 1.12%, respectively. Although the authors reported lower antioxidant values upon comparison with ours, they still successfully prepared silver nanoparticles. The TPC values reported by Olteanu et al. [57] were 389.83 ± 5.45 for lavender and 664.32 ± 6.78 for oregano, determined in mg/mL. Our results suitably correlate with those reported by the authors. Furthermore, *B. nigra* radical scavenging activity and total phenolic content were studied by Lee et al. [58], and they reported the highest values of TPC for the aqueous extract but, on the other hand, the lowest EC_50_ for DPPH scavenging activity, upon comparison with the other solvents. In our case, *B. nigra* seeds represent the second weakest antioxidant. The lowest values of TPC (0.234 mg GAE/mL) and antioxidant capacity (61.78%) were determined for *B. vulgaris* root extract. For *B. nigra*, the DPPH antioxidant activity was expressed as an IC_50_ value of 63.09 ± 1.25 µg/mL and TPC was 6.67 mg GAE/g in literature [59].

There are several methods of evaluating antioxidant activity [60], including an innovative method using the intensity of surface plasmon resonance (SPR). This method assesses the relationship between the antioxidant capacity of phenolic compounds and corresponding optical response [55].

### 3.2. Synthesis and Optical Studies of AgNPs

**Figure 1:**
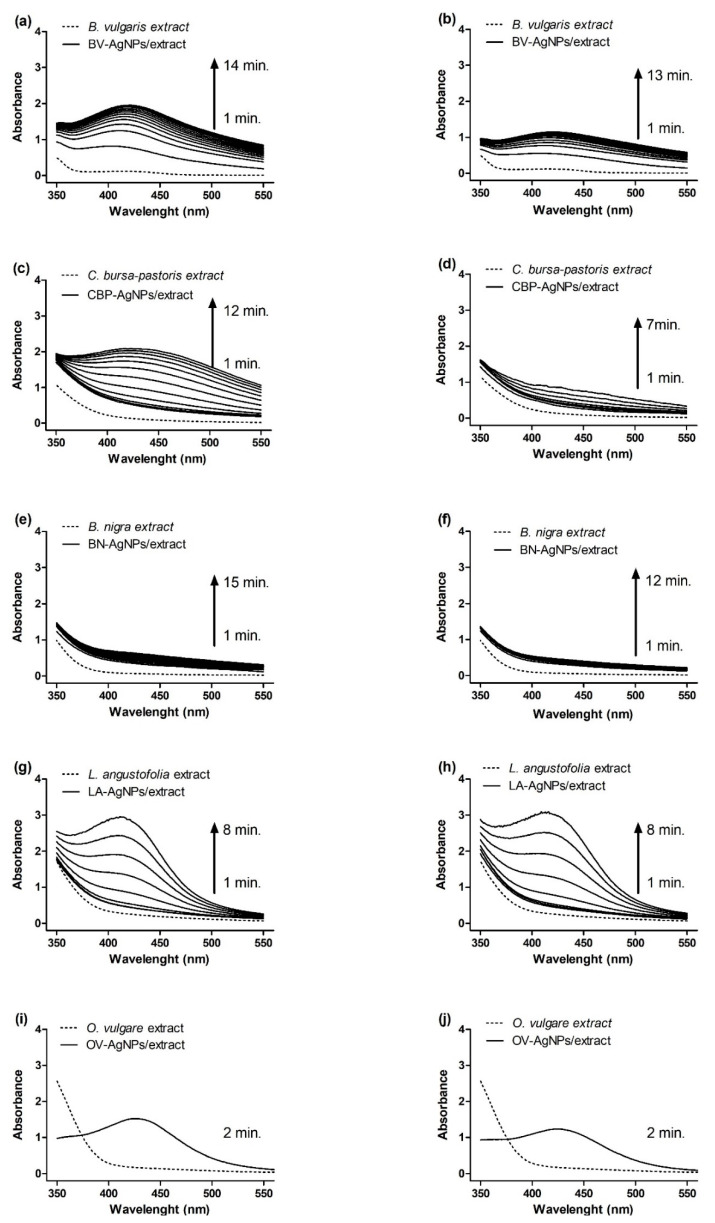
UV-Vis spectra of plant extracts (dash line) and biosynthesised silver nanoparticles (AgNPs) (solid line) prepared from 2 mM (**a**,**c**,**e**,**g**,**i**) and 5 mM AgNO_3_ (**b**,**d**,**f**,**h**,**j**).

The bio-reduction of silver ions to nanosized Ag^0^ was aided by aqueous plant extracts of *B. vulgaris* (BV, root extract), *B. nigra* (BN, seed extract), *C. bursa-pastoris* (CBP, leaves extract), *L. angustifolia* (LA, leaves extract) and *O. vulgare* (OV, leaves extract) as the reducing agents. The 1:9 ratio (extract/AgNO_3_) was required to achieve particles with spherical morphology according to the literature [31]. The primary detection method of bio-reduction of Ag^+^ ions to colloidal nanoparticles (AgNPs) was observed by a visual colour change and confirmed by UV-Vis spectral analysis (Figure 1). The colours of the prepared nanoparticles suspensions were yellow (BN-AgNPs), orange (CBP-AgNPs), greenish-brown (BV-AgNPs) and red (LA-AgNPs and OV-AgNPs).

The light absorption pattern of the extracts and mixture of extracts and silver nitrate, of corresponding concentrations, was monitored in the range of 350–550 nm with a scan step of 1 nm (Figure 1). The formation of AgNPs can be monitored by the increasing intensity of the absorbance band corresponding to the SPR of AgNPs. The UV-Vis spectra showed maximum absorbance between 412 and 426 nm, exhibiting a slight red shift with the time of incubation. Then, the heating was stopped after reaching the maximum absorption (Table 3). The reaction time of Ag^+^ ion reduction is temperature-dependent, and therefore, the enhancement of the reaction temperature increases the reduction rate and shortens the reaction time necessary for AgNP synthesis [61]. The use of higher temperatures was necessary for concentrations of 2 and 5 mM AgNO_3_ to compete within a few minutes (2–15 min). In the case of the lower concentration, the process was slower and, therefore, a temperature of 90 °C was used (in order to accelerate the reaction). On the other hand, at a precursor concentration of 5 mM, the reaction proceeded faster, so it was satisfactory to use a temperature of 80 °C.

In the case of CBP-AgNPs and BN-AgNPs, almost no signal was detected in the monitored region. Interestingly, a weak peak located at 422 nm was observed in the case of CBP-AgNPs (2 mM), whereas for 5 mM, it was not observed. During the aggregation of the AgNPs, metal particles become electronically coupled and this system has a different SPR than individual nanoparticles [62]. The plasmon resonance of nanoparticle aggregates is red-shifted to higher wavelengths, and this shift becomes larger as the NP diameter increases [63]. In our case, the broad absorption spectra of colloid AgNPs were observed in the case of BN-AgNPs and CBP-AgNPs. The formation of aggregated NPs was confirmed by TEM, ZP and PCCS. A visible peak located at 421 nm was registered in the case of BV-AgNPs, and very intensive peaks were exhibited at 412 nm for LA-AgNPs. The UV-Vis spectra of all samples could be easily measured except for OV-synthesised AgNPs. The absorbance of the latter involved a strongly absorbing extract, but after dilution of prepared suspension in the ratio 1:7 with water, a maximum absorbance was observed at ca. 426 nm. Similarly, an increase in absorbance intensity was observed with reaction time corresponding to the observation of AgNP green synthesis using *O. vulgare* [31].

### 3.3. Structural Characterisation of AgNPs Obtained from 5 mM AgNO_3_

In order to obtain information on the NP size, shape, aggregation state, monodispersity in colloidal solutions and localisation of NPs in the matrix [64], a set of characterization techniques was applied. This was done for AgNPs obtained from 5 mM AgNO_3_, as the amount of AgNPs is higher in this case.

**Figure 2:**
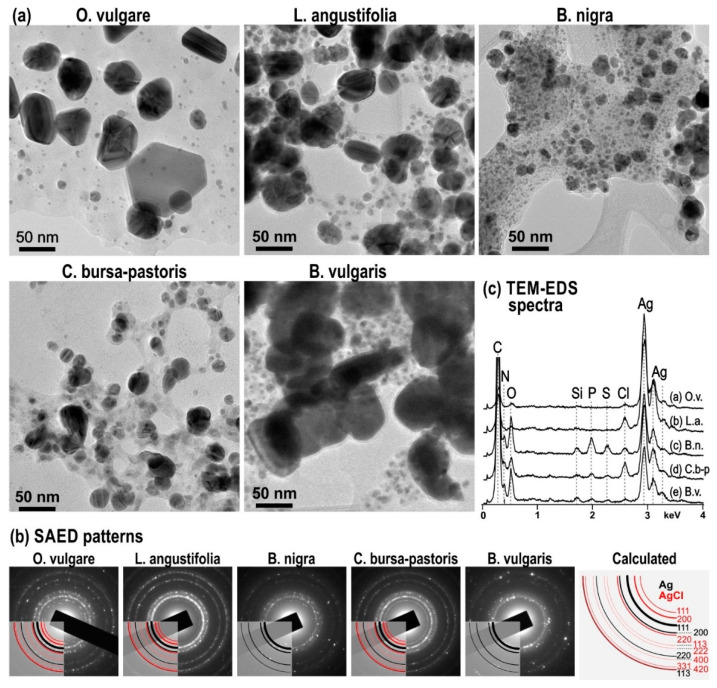
(**a**) The transmission electron microscopy (TEM) images of AgNPs in samples synthesised with different plant extracts from 5 mM AgNO_3_, (**b**) selected area electron diffraction (SAED) patterns of samples and calculated diffraction rings for Ag (black) and AgCl (red), and (**c**) TEM-EDS (energy-dispersive X-ray spectroscopy) spectra taken across areas of different samples including AgNPs and capping agents.

TEM analyses in combination with selected area electron diffraction (SAED) confirmed the formation of AgNPs in all samples with the addition of different plant extracts (Figure 2). SAED patterns in the form of diffraction rings confirm that the samples contain randomly oriented particles with sizes in the nanometre range. While the larger NPs contribute to the brighter diffraction dots, the finer ones yield those with lower brightness. A larger number of finer nanoparticles in the area selected for the diffraction analysis results in the formation of continuous rings as observed, e.g., in the sample *L. angustifolia* in Figure 2b. An additional factor that contributes to the appearance of diffraction pattern is the thickness of the sample; in this case, the organic matrix is quite thick, which weakens the diffraction intensity from smallest NPs. This effect may be observed in the sample synthesised with the addition of *B. nigra*, where diffuse rings from the organic matrix are present in the background of the diffraction pattern, whereas the diffraction dots from the crystalline sample are less strong. The results of SAED analyses revealed that the samples synthesised in the presence of *O. vulgare*, *L. angustifolia* and *C. bursa-pastoris* contain fine AgCl NPs in addition to elemental Ag. Figure 2b also shows the calculated diffraction of diffraction rings from both phases and the d-values of diffraction rings from different lattice planes of both phases (Ag and AgCl), and their relative intensities are listed in Table 4. The formation of the AgCl phase is related to the presence of Cl in the plant-originated capping agents as revealed by TEM-EDS shown in Figure 2c. EDS analyses reveal that, besides chlorine, the organic matrix of *B. nigra*, *C. bursa-pastoris* and *B. vulgaris* also contains other elements such as sulphur, phosphorous, silicon and nitrogen. The presence of nitrogen might result from incomplete reduction of silver nitrate in the initial solution or their presence in the matrix of these plants.

The common feature of all samples is bimodal size distribution of AgNPs. The average size of the finer fraction is typically in the range of ≈5 nm or lower and indicates rapid nucleation of silver on the surface of the organic matrix at the beginning of the reduction process. These fine particles, represented by finer dots, indicate the presence of nuclei available for further growth of AgNPs to several times their size. The average size of coarser crystallites is different in the samples with the addition of different plant extracts, as evident from the TEM images and the results of the measurements given in Table 5. The large AgNPs usually develop as truncated octahedrons (this is especially for NPs prepared using *O. vulgare*). The morphology of the finer NPs is spherical without well-developed crystal faces. The presence of different size distributions of AgNPs is due to the involvement of various biomolecules in capping and bio-reduction of the AgNO_3_ solution. In studies using the same plants we used [29,30,37], TEM micrographs were not provided with the exception of some recent works in which *O. vulgare* was used [31,65]. Therefore, it is quite hard to discuss the results with those papers, as different methods were used for the observation of NP size and these are very often not in agreement (see, e.g., [31]).

Nevertheless, an approximate comparison is possible. In the case of the *O. vulgare* plant (measurement was performed by the DLS method), a bimodal distribution with the smaller fraction of 20 nm and the largest of 200 nm was observed. The average particle size was 136 nm [30]. It follows that the prepared NPs within the discussed study were quite large, similar to our study. When the results obtained directly from the TEM are considered from the two papers using this plant [31,65], the bimodal distribution was observed in both cases. Namely, the average sizes of the AgNPs prepared using the high-energy ball milling method were 3 and 34 nm for smaller and larger fractions, respectively [65]. Upon the classical green approach, the NP sizes were 38 nm and 7 nm for the larger and smaller fractions, respectively [31]. Our results obtained for this plant are in accordance with those papers, also from the EDS analysis point of view, as chlorine was also detected in our case.

Regarding the other plants, namely, *L. angustofolia* and *B. nigra*, the size distribution using different methods (SEM and nanoparticles tracking analysis (NTA)) showed that the particles are finer than in the case of *O. vulgare*, which is also in accordance with our study. In the case of *Lavandula*, the maximum was evidenced around 13 nm and particles larger than 40 nm could not be detected [37]. In the case of *B. nigra*-based synthesis reported in [29], only the average size of 41 nm was mentioned. Regarding the shape of all NPs, these can be very rich depending on the plant used; however, the fact that the larger particles have clearer geometry and that the smaller tend to be spherical was evidenced in the TEM, at least for the *O. vulgare* [31,65].

**Figure 3:**
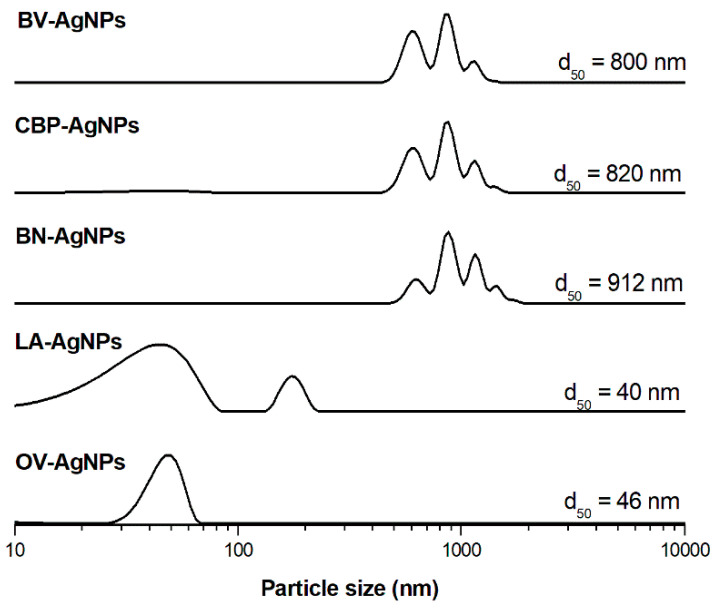
Grain size distribution of biosynthesised AgNPs from 5 mM AgNO_3_ determined by PCCS.

In order to investigate the effect of the material responsible for the reduction from different plants and its interaction with the formed AgNPs, the ZP and PCCS measurements were performed. The results are presented in Table 6 and Figure 3, respectively. The individual nanoparticles are prone to forming agglomerates or, at least, to being connected to larger grains by the residual organic matrix from the plant extracts. These grains can be registered also by the PCCS method, and the stability of the prepared suspensions can be estimated by ZP measurements.

The smallest unimodal grains were obtained by using the extract of *O. vulgare*, where the average hydrodynamic diameter (d_50_) of obtained grains was 46 nm. The ZP of this nanosuspension was estimated as −11.9 mV at pH 3.20. All other suspensions contain grains with different sizes, starting from several nanometres and ending in huge aggregates with the size of several micrometres. The AgNPs prepared from *L. angustifolia* for 3 min exhibit d_50_ value 40 nm, and the values of ZP and pH are −15.8 mV and 3.31, respectively. The aggregation is manifested in larger-size grain formation according to PCCS analysis. The average grain size for the BN-AgNPs sample was estimated at 912 nm with a value of ZP of −6.5 mV at pH 4.28 and, for the *C. bursa-pastoris*, it was 820 nm with a ZP value of −18.1 mV at pH 4.81. The 800 nm grains were obtained using *B. vulgaris* with a very low ZP value of 0.38 nm at pH 3.77, which is a sign of a very unstable suspension with the tendency of particles to coagulate. Regarding ZP measurements, this technique has already been used for evaluation of the interaction between the organic matrix and AgNPs in papers dealing with their synthesis using *O. vulgare* [30,31]. In both cases, negative values were detected, which is in accordance with our observations and documents the quite high stability of the nanosuspension. The negative values of the ZP of all samples except BN-AgNPs may result from negatively charged compounds surrounding the surface of nanoparticles [66].

The grain size distribution (Figure 3) documents the differences between the matrices; mainly two groups can be distinguished: (i) *Origanum* and *Lavandula*, which provide quite unimodal and well-dispersed grains, and (ii) the rest of the plants, in which quite agglomerated particles can be detected. This result correlates with the amount of TPC, which are possibly substances responsible for the reduction in the NPs. Plants with a higher content of phenolic substances provide nanoparticles of smaller size. It is possible that, in the first group, they are quite homogeneously distributed whereas, in the second case, they are scarcer, but many of them are present at one spot and, therefore, the significant reduction occurs only at some locations.

It has to be noted that, if the results of the PCCS method and TEM are compared, they are almost completely opposite (TEM: BV-AgNPs and OV-AgNPs have larger particles, while the other plants have small particles; PCCS: LA-AgNPs and OV-AgNPs have smaller grains, while the other plants have large grains). This observation underlines the fact that the results of PCCS and TEM measurements cannot be compared whereas the former method characterises the size of individual NPs and the latter characterises the agglomerated grains.

**Figure 4:**
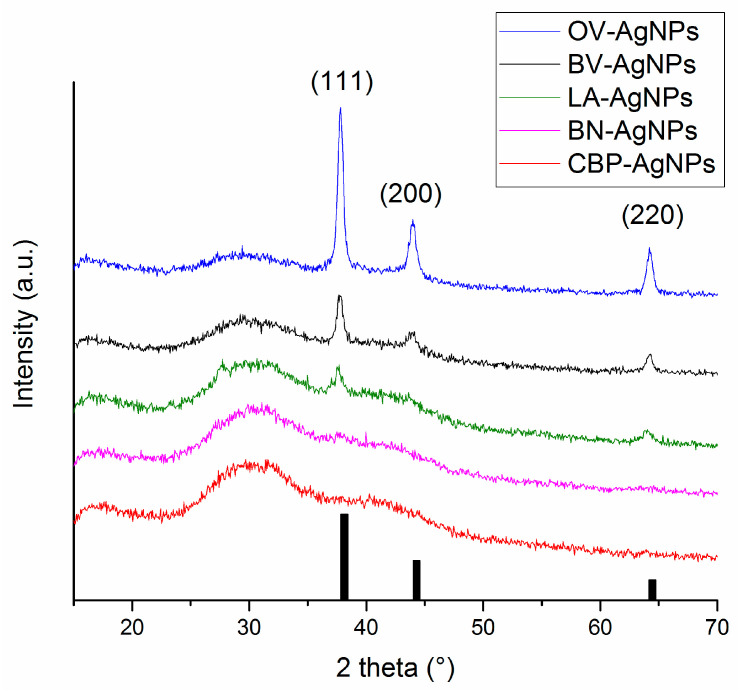
The X-ray diffraction (XRD) patterns of the dried solids after centrifugation after the green synthesis of AgNPs starting from 5 mM AgNO_3_ and using 5 different plants. Bar graphs represent the Bragg‘s reflections of cubic base-centred (fcc) elemental silver (ICDD-PDF2-065-2871). The Miller indices of the crystallographic planes corresponding to the three most intensive reflections of fcc-Ag are indexed in parentheses.

The XRD patterns of the powders obtained after centrifugation are provided in Figure 4. The reflections corresponding to elemental silver have been found in just three out of five products. Namely, the presence of elemental silver in the solid residues after centrifugation is undeniable for OV-AgNPs, BV-AgNPs and LA-AgNPs. For these systems, the largest crystallite size was determined from TEM (Table 5), and the SPR peak could be well-identified in Figure 1i,j. For the systems where the produced nanoparticles were apparently smaller (CBP-AgNPs and BN-AgNPs) with average crystallite size below 20 nm according to TEM (see Figure 2a and Table 5) and the SPR peak hardly found (Figure 2c–f), the reflections corresponding to elemental silver are absent. As the produced nanoparticles in these cases are very fine, it is highly probable that a significant amount of them were dispersed in the supernatant and remained there despite intensive centrifugation and, thus, could not be detected in the solid residue. Moreover, smaller NPs exhibit much broader and diffuse peaks, so their signal also might have been lost in the noise in the last two cases.

**Figure 5:**
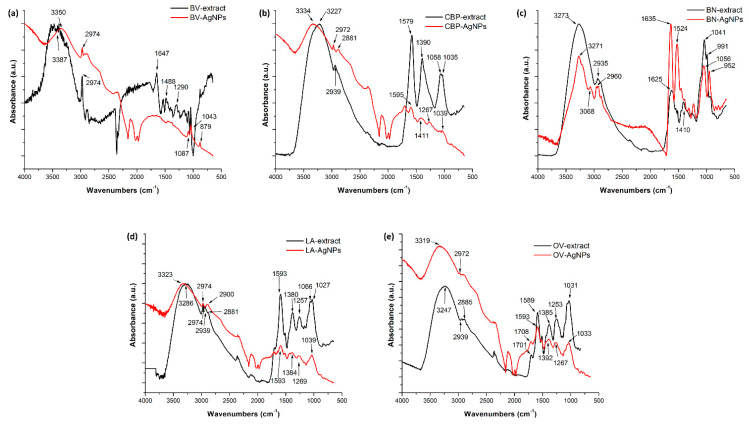
Attenuated total reflectance (ATR)-FTIR absorption spectra of plant extracts and synthesised AgNPs from 5 mM AgNO_3_, (**a**) BV-AgNPs, (**b**) CBP-AgNPs, (**c**) BN-AgNPs, (**d**) LA-AgNPs and (**e**) OV-AgNPs.

FTIR analysis of the dried plant extracts and prepared AgNPs was performed, and the results are shown in Figure 5. From the results obtained, the FTIR spectra of the plant extracts and as-synthesised nanoparticles were very similar with some slight shifts. The functional groups of compounds were identified, where OV-AgNP showed peaks at 3319, 2972, 1593, 1392, 1267, 1060 and 1033 cm^−1^, whereby the extract main peaks were 3247, 2939, 1589, 1388, 1253, 1060 and 1031 cm^−1^. The FTIR peaks around 3319 and 3247 were attributed to O–H stretching vibrations, and the peak at 2972 and 2939 were attributed to C–H stretching vibrations, which are present according to the presence of the polyphenolic compound (TPC content in Table 2) and other compounds such as the carbohydrates of amides. Moreover, stretching vibrations of C=C at 1593 and 1589 cm^−1^ and of −C−O− at 1060 cm^−1^ were observed in both spectra. Polyphenols are the main phytochemicals contributing to in metallic reduction [66]. The −C−H bending vibrations were found at 1392 and 1388 cm^−1^. These crucial peaks were also found in all the studied samples of the prepared NPs, as shown in Figure 5. Consequently, the extracted compound plays a dual role in biosynthesis in addition to the reducing function and the stabilising and capping function. A stabilising coating over the synthesised AgNPs can be used for future functionalisation of nanoparticles, for example, as a drug carrier.

### 3.4. Antibacterial Activity of AgNPs

**Figure 6:**
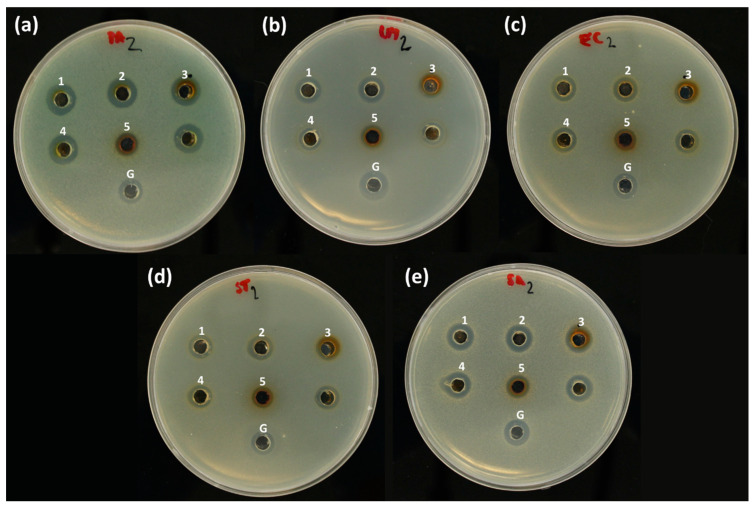
Petri dishes showing the antimicrobial activity of 1. BN-AgNPs, 2. BV-AgNPS, 3. LA-AgNPs, 4. CBP-AgNPs and 5. OV-AgNPs prepared from 5 mM AgNO_3_ and gentamicin sulphate (G) on (**a**) *P. aeruginosa*, (**b**) *L. monocytogenes*, (**c**) *E. coli*, (**d**) *S. enterica* ser. Typhimurium and (**e**) *S. aureus.*

Silver and its compounds are well-known as some of the most universal antibacterial substances against a wide range of microorganisms from different classes. In this study, the antibacterial activity of the biogenic AgNPs against pathogenic bacteria was employed using the agar well-diffusion method on five types of bacteria: *L. monocytogenes*, *S. aureus* (Gram-positive), *P. aeruginosa*, *E. coli* and *S. enterica* ser. Typhimurium (Gram-negative). Sample pictures of Petri dishes containing agar, bacterial colonies and punched holes with AgNP-containing nanosuspensions after 24 h incubation can be seen in Figure 6. All the tested bacteria were resistant to plant extracts equally diluted with water (*p* < 0.001; see Table S1 in the Supplementary Materials). Additionally, silver nitrate (2 mM and 5 mM) exhibited significant antibacterial activity. The obtained results for the final concentrations 2 mM and 5 mM of the precursor are presented in Table 7 and Table S1. The values obtained for 10 mM gentamicin sulphate as a positive control are considered 100% RIZD.

The antibacterial study showed that AgNPs prepared using different plant extracts and AgNO_3_ (2 mM and 5 mM) possessed antibacterial activity. It is apparent from the results that the AgNPs prepared by employing plant extracts display different antibacterial activities toward various strains of bacteria. In general, an antibacterial activity similar to standard gentamicin sulphate (10 mM) was observed in all studied samples except AgNPs prepared from 2 mM AgNO_3_ using *O. vulgare* (*p* < 0.05). On the other hand, AgNPs prepared from 5 mM AgNO_3_ with the same extract showed antibacterial activity similar to the positive control 10 mM gentamicin sulphate. The most effective were AgNPs prepared using the extract of *Brassica nigra*, where the finest fraction of AgNPs was found (according to TEM analysis). This is in correlation with the finding that antimicrobial activity depends on the size and shape of AgNPs [67]. In general, the antibacterial activity of the NPs prepared from 5 mM AgNO_3_ was higher than that of the NPs prepared from 2 mM AgNO_3_, as evidenced from Table 7. Dependence of the antibacterial activity on the concentration of AgNPs was also reported for multiple bacteria by Sankar et al. [30]. The most resistant bacteria to all types of prepared AgNPs was *L. monocytogenes*. It could be based on the morphological structure of the bacterial cell wall because *L. monocytogenes* is a Gram-positive bacteria. The lower activity on Gram-positive bacteria was reported also by other researchers [2,68].

On the contrary, easier penetration in the case of Gram-negative bacteria is responsible for the higher susceptibility compared with the Gram-positive bacteria [69]. In the case of *P. aeruginosa*, nanoparticles prepared from 5 mM AgNO_3_ using plant extracts from *C. bursa-pastoris*, *B. nigra*, *L. angustifolia* and *B. vulgaris* exhibited higher activity than the control antibiotic gentamicin sulphate (100%). In general, it can be concluded that the biosynthetically prepared AgNPs from different plant extracts are effective antibacterial agents and that the observed effect has been concentration-dependent. Similar results show that nanoparticles were prepared from an aqueous extract of *B. nigra* seeds whereas 1 mM AgNO_3_ did not show activity against tested bacteria *Propionibacterium acnes*, *Pseudomonas aeruginosa* and *Klebsiella pneumoniae* [29]. A previous report of AgNPs prepared from lavender leaves shows antibacterial activity against *Escherichia coli*, *Pseudomonas aeruginosa*, *Proteus mirabilis*, *Bacillus cereus*, *Klebsiella oxytoca*, *Salmonella typhi* and *Staphylococcus aureus* [69].

In conclusion, the antibacterial activity of AgNPs is dependent on three factors: the concentration of AgNPs, the type of plant and the type of bacteria. The antibacterial activity of AgNPs prepared from plant extracts is much higher than the antibacterial activity of the corresponding extract, which showed no activity (Table S1). The enhancement of the activity is most likely based on the silver ion, released by AgNPs, destroying cell walls and secondary metabolites adsorbed on AgNPs [69]. It should be noted that the resulting antimicrobial activity can also be affected by silver ion activity in the form of produced AgCl or unreacted AgNO_3_. Bioactive compounds in the organic matrix could also be responsible for the increased antibacterial activity of AgNPs. They may have improved the ability to interact with the cell wall.

## 4. Conclusions

We successfully showed the universality of the proposed simple green synthetic approach for the synthesis of AgNPs with different plant extracts of *B. vulgaris* (root), *B. nigra* (seeds), *C. bursa-pastoris* (leaves), *L. angustifolia* (leaves) and *O. vulgare* (leaves). Herein, the presence of elemental Ag was confirmed in most cases by measuring the XRD. The green synthesis proceeded in a very rapid manner, as it was finished within 15 min for all plants at elevated temperatures. Synthesis is accelerated and simplified, particularly as it takes place directly in the cuvette.

This investigation provides evidence about the influence of plant diversity in the composition of silver nanoparticle biosynthesis. A positive correlation between the TPC, antioxidant activity and biosynthesis rate have been identified. A lower antioxidant activity and TPC resulted in slower reaction rate and final agglomeration of very fine nanoparticles (according to PCCS). Additionally, the SPR formation of AgNPs is highly dependent on size, shape, surrounding dielectric properties and most significantly on aggregation. In general, larger particles were prepared when using *Origanum* and *Lavandula* plants and smaller-forming aggregates were prepared in the case of the other plants. The FTIR spectra proved the successful interaction between the produced Ag nanoparticles and biological compounds from plants, most probably phenolics, amides or carbohydrates. The prepared NPs exhibited, in TEM measurements, two types of morphology: smaller spherical and larger truncated octahedral shapes. Finally, the synthesised AgNPs exhibited significant antimicrobial activity against both Gram-positive and Gram negative bacteria.

## Figures and Tables

**Table 1:** Summary table of the main findings of the studies performing green synthesis using the selected plants.

Plant Species	Concentration of AgNO_3_	Characterisation	Activity	Size of AgNPs (nm)	Shape of AgNPs	Ref.
*B. nigra*	1 mM	UV-Vis	antibacterial	41	-	[29]
		NTA				
		FTIR				
		DLS				
*O. vulgare*	1 mM	UV-Vis	antibacterial	63–85	spherical	[30]
		FTIR				
		FE-SEM				
		XRD				
*O. vulgare*	0.5 mM	UV-Vis	antibacterial	2–25	spherical	[32]
		XRD				
		TEM				
		EDX				
		FTIR				
*O. vulgare*	1, 2.5, 5, 10 and 100 mM	UV-Vis	antibacterial	151–4819	polygonal	[33]
		PCCS				
		TEM				
*B. vulgaris*	0.5, 1, 3 and 10 mM	UV-Vis	antibacterial	30–70	spherical	[34]
		TEM				
		XRD				
		DLS				
*L. angustifolia*	10 mM	UV-Vis	-	20	spherical, triangular	[35]
		SEM				
		XRD				
		TEM				
		EDS				
*L. angustifolia* fractions	1 g/L	UV-Vis	cytotoxic	35.4 ± 1.6 and 56.4 ± 2.4	round- shape, tetrahedron, polyhedron	[36]
		DLS				
		SEM				
*L. angustifolia*	1 mM	UV-Vis	antioxidant	10–30	spherical	[37]
		EDXRF				
		SEM				
*B. vulgaris*	2 and 5 mM	UV-Vis	antibacterial antioxidant	75.7 ± 17.1	spherical	This study
*B. nigra*		TEM		14.7 ± 7.9	spherical	
*C. bursa-pastoris*		ZP		16.2 ± 8.4	spherical	
*L. angustifolia*		PCCS		37.8 ± 10.7	spherical	
*O. vulgare*		EDS		46.1 ± 19.7	smaller-spherical, larger-truncated octahedral	

UV-Vis—ultraviolet and visible spectroscopy, FTIR—Fourier transform infrared spectroscopy, PCCS—photon cross-correlation spectroscopy, NTA—nanoparticles tracking analysis, TEM—transmission electron microscopy, FE-SEM—field emission-scanning electron microscopy, ZP—zeta-potential, XRD—X-ray diffraction, EDS—energy-dispersive X-ray spectroscopy, DLS—dynamic light scattering, EDXRF—energy dispersive X-ray fluorescence.

**Table 2:** The 2,2-diphenyl-1-picrylhydrazyl (DPPH) antioxidant activity and total phenolic content (TPC) of plant extracts.

Plant Extract	TPC (mg GAE/mL) ± SD	DPPH (%) ± SD
*Berberis vulgaris*	0.234 ± 0.009	61.78 ± 1.80
*Brassica nigra*	0.297 ± 0.028	73.77 ± 3.47
*Capsella bursa-pastoris*	0.464 ± 0.031	89.68 ± 3.18
*Lavandula angustifolia*	0.884 ± 0.022	92.97 ± 0.04
*Origanum vulgare*	4.569 ± 0.766	94.98 ± 0.08

**Table 3:** The surface plasmon resonance (SPR) peak position and reaction time for corresponding plant extract/AgNO_3_ mixtures.

Plant-AgNPs	SPR Peak Position (nm)	Time (min) 2/5 mM AgNO_3_
BV-AgNPs	421 nm	14/12
CBP-AgNPs	422 nm	12/7
BN-AgNPs	Not found	15/12
LA-AgNPs	412 nm	8/8
OV-AgNPs	426 nm	2/2

**Table 4:** Relevant structural data for determination of Ag (ICDD-PDF2-065-2871) and AgCl (ICDD-PDF2-085-1355) by SAED.

Phase	Hkl	D (nm)	I/I_0-Ag_ (%)	I/I_0-AgCl_ (%)
AgCl	111	3.2037		51.3
AgCl	200	2.7745		100.0
Ag	111	2.3592	100.0	
Ag	200	2.0431	48.2	
AgCl	220	1.9618		67.0
AgCl	113	1.6730		23.0
AgCl	222	1.6018		22.3
Ag	220	1.4447	29.8	
AgCl	400	1.3872		10.5
AgCl	331	1.2730		10.0
AgCl	420	1.2407		31.8
Ag	113	1.2326	29.8	

**Table 5:** Average diameter of AgNPs in samples with different plant extracts determined from TEM.

Sample	Coarse Fraction
OV-AgNPs	46.1 ± 19.7 nm
LA-AgNPs	37.8 ± 10.7 nm
BN-AgNPs	14.7 ± 7.9 nm
CBP-AgNPs	16.2 ± 8.4 nm
BV-AgNPs	75.7 ± 17.1 nm

**Table 6:** Zeta potential and pH values of synthesised AgNPs from 5 mM AgNO_3_.

Sample	Zeta Potential (mV)	pH
OV-AgNPs	−11.9	3.20
LA-AgNPs	−15.8	3.31
BN-AgNPs	−6.5	4.28
CBP-AgNPs	−18.1	4.81
BV-AgNPs	0.38	3.77

**Table 7:** Antibacterial activity of biosynthesised AgNPs.

Plant Extract-AgNPs	Bacteria Type	*P. aeruginosa*	*L. monocytogenes*	*E. coli*	*S. enterica* ser. Typhimurium	*S. aureus*
	Conc. of AgNO_3_	RIZD (% ± SD)				
CBP-AgNPs	2 mM	92.61 ± 10.07	55.17 ± 12.40	80.76 ± 2.94	80.10 ± 4.72	96.03 ± 5.90
	5 mM	148.33 ± 1.50	59.88 ± 4.15	87.78 ± 5.11	98.16 ± 8.63	102.27 ± 5.88
BN-AgNPs	2 mM	73.87 ± 12.45	60.65 ± 1.80	83.31 ± 3.45	77.33 ± 7.25	93.12 ± 14.77
	5 mM	152.53 ± 8.60	84.44 ± 9.21	99.09 ± 15.35	99.05 ± 5.72	104.80 ± 4.35
LA-AgNPs	2 mM	97.78 ± 12.20	21.79 ± 15.48	82.77 ± 1.03	82.31 ± 2.82	89.91 ± 4.78
	5 mM	146.25 ± 10.62	80.06 ± 3.33	93.81 ± 12.41	97.25 ± 12.05	101.29 ± 6.33
BV-AgNPs	2 mM	77.57 ± 0.97	72.18 ± 8.99	89.21 ± 6.48	88.78 ± 9.04	93.64 ± 7.07
	5 mM	151.12 ± 18.28	83.67 ± 6.47	94.99 ± 5.14	99.69 ± 18.35	101.93 ± 8.33
OV-AgNPs	2 mM	0.00 ± 0.00 *	23.73 ± 2.93 *	0.00 ± 0.00 *	0.00 ± 0.00 *	0.00 ± 0.00 *
	5 mM	97.75 ± 2.05	58.84 ± 7.38	71.53 ± 8.95	66.61 ± 7.05	74.14 ± 5.59
AgNO_3_	2 mM	96.92 ± 19.44	67.80 ± 10.74	89.25 ± 8.02	86.25 ± 9.24	90.66 ± 6.34
	5 mM	111.37 ± 10.52	90.47 ± 12.41	99.78 ± 4.84	104.00 ± 7.27	117.81 ± 2.11

* The results are statistically significant at the level p < 0.05; other values are not statistically significant.

## Data Availability

Not applicable.

## Supplementary Materials

The following are available online at https://www.mdpi.com/article/10.3390/nano11041005/s1, Table S1: The mean inhibition zone diameter (IZD) ± SD of plant extracts and prepared silver nanoparticles (AgNPs) of 3 replicate plates.
