# Supplementary material for: Green Synthesis of Silver Nanoparticles with Antibacterial Activity Using Various Medicinal Plant Extracts: Morphology and Antibacterial Efficacy
Source: Nanomaterials (Basel). 2021 Apr 14;11(4):1005. doi: 10.3390/nano11041005 (PMC8070782; doi:10.3390/nano11041005)
Supplement: Supplementary file 1 [file nanomaterials-11-01005-s001.pdf]

SUPPLEMENTARY INFORMATION

Table S1. The mean inhibition zone diameter (IZD) ± SD of plant extracts and prepared silver nanoparticles (AgNPs) of 3 replicate plates.

	**Bacteria type**	*P. aeruginosa*	*L. monocytogenes*	*E. coli*	***S. enterica* ser. Typhimurium**	*S. aureus*
	Conc. of $AgNO_3$	IZD ± SD (mm)	IZD ± SD (mm)	IZD ± SD (mm)	IZD ± SD (mm)	IZD ± SD (mm)
CBP extract	-	5.50 ± 0.00	5.50 ± 0.00	5.50 ± 0.00	5.50 ± 0.00	5.50 ± 0.00
CBP-AgNPs	2 mM	11.80 ±2.01	9.23 ± 0.60	9.30 ± 0.23	8.60 ± 0.	10.78 ± 0

					27	.23
	5 mM	12.57 ± 0.33	9.55 ± 0.06	10.19 ± 0.45	9.97 ± 0.31	11.39 ± 0.36
BN extract	-	5.50 ± 0.00	5.50 ± 0.00	5.50 ± 0.00	5.50 ± 0.00	5.50 ± 0.00
BN-AgNPs	2 mM	10.49 ± 0.84	9.82 ± 0.31	9.36 ± 0.21	8.89 ± 0.19	10.51 ± 0.63
	5 mM	12.77 ± 0.21	11.21 ± 0.54	10.76 ± 0.57	10.03 ± 0.50	11.53 ± 0.22
LA extract	-	5.50 ± 0.00	5.50 ± 0.00	5.50 ± 0.00	5.50 ± 0.00	5.50 ± 0.00
LA-AgNPs	2 mM	12.07 ± 1.86	7.01 ± 1.06	9.40 ± 0.23	8.68 ± 0	10.44 ±

					.12	0.27
	5 mM	12.47 ± 0.25	10.92 ± 0.39	10.51 ± 0.69	9.92 ± 0.32	11.33 ± 0.39
BV extract	-	5.50 ± 0.00	5.50 ± 0.00	5.50 ± 0.00	5.50 ± 0.00	5.50 ± 0.00
BV-AgNPs	2 mM	10.74 ± 0.07	10.61 ± 0.16	9.63 ± 0.24	9.40 ± 0.22	10.54 ± 0.20
	5 mM	12.68 ± 0.32	11.15 ± 0.19	10.56 ± 0.18	10.03 ± 0.72	11.37 ± 0.51
OV extract	-	-	-	-	-	-
OV-AgNPs	2 mM	5.50 ± 0.00	7.18 ± 0.14	5.50 ± 0.00	5.50 ± 0.00	5.50 ± 0.00
	5 mM	10.17 ± 0.33	9.47 ± 0.34	9.31 ± 0.47	8.5	9.7

					4 ± 0.29	7 ± 0.38
gentamicin sulphate		10.28 ± 0.39	12.27 ± 0.35	10.84 ± 0.37	10.07 ± 0.24	11.26 ± 0.08
		12.25 ± 2.49	12.64 ± 0.63	10.13 ± 0.17	9.90 ± 0.20	10.89 ± 0.20
		12.44 ± 2.67	12.34 ± 0.62	10.21 ± 0.32	9.36 ± 0.11	11.02 ± 0.56
$AgNO_3$	5 mM	13.02 ± 0.71	11.94 ± 0.97	10.13 ± 0.37	10.07 ± 0.25	11.85 ± 0.22
$AgNO_3$	2 mM	12.04 ± 1.31	10.30 ± 0.45	9.63 ± 0.29	9.29 ± 0.24	10.39 ± 0.31

CBP - *Capsella bursa-pastoris,* BN - *Brassica nigra,* LA - *Lavandula angustifolia,* BV - *Berberis vulgaris* OV - *Origanum vulgare*
